# Real‐World Effectiveness of Sotrovimab in Patients Infected With SARS‐CoV‐2 Omicron Subvariant BA.2 in Western Sydney, Australia

**DOI:** 10.1002/jmv.70235

**Published:** 2025-02-13

**Authors:** Eric Kalo, Ziad Basyouni, Gideon Meyerowitz Katz, Vahid Karkvandi, Leanne Watson, Helen Crowther, Scott Read, Matthew V. N. O'Sullivan, Jasmin Ellis, Joanne Medlin, Golo Ahlenstiel

**Affiliations:** ^1^ Blacktown Clinical School and Research Centre, School of Medicine Western Sydney University Blacktown New South Wales Australia; ^2^ Integrated and Community Health, Western Sydney Local Health District, Blacktown Hospital Blacktown New South Wales Australia; ^3^ Blacktown Hospital, Western Sydney Local Health District Blacktown New South Wales Australia; ^4^ Storr Liver Centre The Westmead Institute for Medical Research Westmead New South Wales Australia; ^5^ Centre for Infectious Diseases and Microbiology Westmead Hospital Westmead New South Wales Australia; ^6^ New South Wales Health Pathology ICPMR—Westmead Westmead New South Wales Australia; ^7^ Sydney Infectious Diseases Institute University of Sydney Camperdown Australia

**Keywords:** COVID‐19, Delta, hospitalisation, monoclonal, Omicron BA.2, SARS‐CoV‐2, sotrovimab

## Abstract

Laboratory‐based findings suggest that Sotrovimab is significantly less effective against emerging CARS‐CoV‐2 variants, however, clinical data is lacking. Here we examined the effectiveness of sotrovimab, in preventing emergency department (ED) presentation and subsequent hospitalization in high‐risk subgroups of patients during the SARS‐CoV‐2 Delta and Omicron waves in Western Sydney, Australia (*n* = 515). Risk for ED attendance was comparable in Omicron patients, whether BA.1 or BA.2, compared to Delta patients (hazard ratio of 0.97 [0.36–2.64]). These findings highlight the need for caution when using in vitro findings to drive clinical practice, especially when the consequence is to withhold potentially lifesaving treatment.

## 1 Introduction

A confirmed 7.06 million people worldwide have died from the COVID‐19 pandemic caused by severe acute respiratory syndrome coronavirus 2 (SARS‐CoV‐2) as of August, 2024, with global estimates of the total death toll exceeding 14 million as of 2022 [1]. Within Australia, approximately 11.8 million confirmed cases have been reported according to World Health Organization (WHO) [2]. The massive scale and prevalence of the COVID‐19 pandemic combined with limited antiviral therapies have enabled the SARS‐CoV‐2 virus to mutate numerous times into variants of concern that are antigenically distinct from the wild‐type SARS‐CoV‐2. Mutations in these subvariants have been linked to increasing transmission, replicative fitness, immune evasion, and resistance to adaptive immune responses acquired from previous variants or vaccines [3, 4]. Although, COVID‐19 no longer represents a Public Health Emergency of International Concern according to the WHO, the lack of protection against new variants poses significant global threats for the resurgence of breakthrough infections and raises the possibility of COVID‐19 recurrence. Recently, a newly emerged omicron subvariant JN.1 (or BA.2.86.1.1), which is phylogenetically distinct from SARS‐CoV‐2 omicron XBB lineages, has garnered global attention. Since late 2023, it became the prominent circulating variant of interest globally and has been reported by 71 countries.

As novel variants of concern emerge, there remains an urgent unmet need for therapeutic agents that exhibit a high barrier to resistance and remain effective, independent of virus evolution. Monoclonal antibodies (mAbs) are one such therapy that is capable of neutralizing SARS‐CoV‐2 by targeting evolutionarily conserved epitopes such as the SARS‐CoV‐2 receptor‐binding motif that lies outside rapidly evolving loci [5]. The use of mAbs presented as an antiviral therapeutic solution for clinically vulnerable individuals with severe COVID‐19 is of paramount importance in high‐risk individuals where vaccination is contraindicated, the individual is immunocompromised/suppressed or when no vaccine exists that specifically targets novel subvariants [6].

Sotrovimab, formerly known as VIR‐7831, is an engineered human mAb directed against the spike protein of SARS‐CoV‐2 that can neutralise multiple coronavirus strains, including SARS‐CoV‐1 [5]. It additionally drives potent effector functions, including antibody‐dependent cellular cytotoxicity (ADCC) and antibody‐dependent cellular phagocytosis (ADCP) [7]. In a randomized clinical trial (COMET‐ICE, NCT04545060) conducted during the initial period of the pandemic predominated by the original alpha (wild‐type) variant and before rolling out of vaccination programmes, a single intravenous (IV) infusion of sotrovimab (500 mg) was shown to significantly reduce the risk of all‐cause hospitalization (of > 24‐h duration) or death from 6% to 1% (relative risk reducation 79%) compared with placebo in high‐risk patients with COVID‐19 [8]. Consequently, sotrovimab was first granted Emergency Use Authorization (EUA), individually or in combination with other mAbs, in the United States by the FDA (Food and Drug Administration) and other regulatory agencies across the world, including Australia's TGA (Therapeutic Goods Administration). As new variants emerged, the FDA revised and limited the use of sotrovimab due to efficacy concerns [9]. The Omicron BA.2 subvariant that became dominant across the globe in March 2022 demonstrated reduced neutralisation by sotrovimab in vitro [9, 10, 11, 12]. Consequently, the prescription of sotrovimab was then limited across regions with > 50% Omicron BA.2 prevalence.

In the absence of clinical trials assessing the efficacy of sotrovimab against the Omicron BA.2 variant, real‐world data must be used to determine if there is any clinical benefit that may contradict in vitro efficacy interpretation. Here, we use real‐world data to report on the effectiveness of sotrovimab on the risk of emergency presentation visits and hospitalization in patients and high‐risk subgroups during the SARS‐CoV‐2 Delta and Omicron waves in Western Sydney, Australia.

## 2 Methods

This study followed the STROBE reporting guidelines and was approved by the Human Research Ethics Committee of Western Sydney Local Health District (WSLHD) (Approval Number 2022/ETH00507). Informed consent was obtained from each patient included in the study and the study protocol conforms to the ethical guidelines of the Declaration of Helsinki, as reflected in the approval by the WSLHD Human Research Ethics Committee. Between August, 2021 and May, 2022, 515 outpatients with mild/moderate COVID‐19, confirmed using the AllPlex SARS‐CoV‐2 assay (Seegene, RV10248X), of which 174 patient samples were sequenced to detect their SARS‐CoV‐2 strain using the Illumina MiSeq platform (MiSeqMicro Reagent Kit V2, MS‐102‐2002). Demographic and clinical records including comorbidities and number of vaccinations were collected (Tables 1 and S1). Death was identified from the state and hospital death registries. All the patients received Single‐use vial of 500 mg in 8 mL (62.5 mg/mL) concentrated injection for IV infusion of sotrovimab (GlaxoSmithKline and Vir Biotechnology) according to the guidelines outlined in the NSW Therapeutic Advisory Group (TAG) Drug Guideline for sotrovimab (Table S2). The administration of the drug was conducted at WSLHD facilities and patients monitored via daily telehealth for up to 30 days or until negative results by real‐time RT‐PCR were obtained.

**Table 1:** Characteristics of study population.

Characteristic	Delta	OmicronBA.1	OmicronBA.2
*N*	(*n* = 74)	(*n* = 210)	(*n* = 231)
Sequenced	12	81	81
Age	45.3 (15.5)	54.0 (16.9)	51.4 (15.5)
Sex			
Female	35 (47.3%)	96 (45.7%)	126 (54.5%)
Male	39 (52.7%)	114 (54.3%)	105 (45.5%)
Vaccine Status			
Unvaccinated	20 (27.0%)	28 (13.3%)	11 (4.8%)
One dose	34 (45.9%)	3 (1.4%)	3 (1.3%)
Two doses	20 (27.0%)	100 (47.6%)	72 (31.2%)
Three doses	—	77 (36.7%)	125 (54.1%)
Four doses	—	1 (0.5%)	20 (8.7%)
Comorbidities			
Cardiac	9 (12.2%)	41 (19.5%)	24 (10.4%)
Diabetes	16 (21.6%)	54 (25.7%)	47 (20.3%)
Chronic Lung Disease	10 (13.5%)	23 (11.0%)	27 (11.7%)
Kidney Disease	2 (2.7%)	68 (32.4%)	49 (21.2%)
Liver Disease	—	4 (1.9%)	4 (1.7%)
Cancer	3 (4.1%)	32 (15.2%)	40 (17.3%)
Immunocompromised	12 (16.2%)	136 (64.8%)	173 (74.9%)
Obese (BMI > 30)	43 (58.1%)	45 (21.4%)	35 (15.2%)

COVID‐19 related emergency department (ED) presentation and admissions were stratified by variant and demonstrated using standard Kaplan–Meier plots. The influence of confounding factors was assessed using a Cox regression controlling for comorbidities, age, sex, and vaccine doses (Table S3). These potential confounders were selected as they were considered the most likely to have a plausible effect on the likelihood of hospital admission.

## 3 Results

Sotrovimab was administered according to the clinical criteria for administration in New South Wales, Australia. Specifically, sotrovimab was administered within 5 days of symptom onset to patients > 55 years of age, who were not fully vaccinated, who did not require oxygen and possessed at least one risk factor for disease progression (Table S2). Assessment of sotrovimab efficacy was performed on the entire cohort of patients that received sotrovimab (*n* = 515), as well as the subgroup of patients for whom the variant was confirmed by sequencing (*n* = 174). Analysis of the full cohort was performed using publicly available data on http://covariants.org/ estimating the prevalence of SARS‐CoV‐2 variants in Australia from August 2, 2021 to May 23, 2022. The month during which a given patient was diagnosed with SARS‐CoV‐2 was used as a proxy to identify the predominant circulating variant, which was subsequently assigned to the patient (Figure S1).

The predominant circulating SARS‐CoV‐2 variant (> 75% prevalence) was Delta (or B.1.617.2) from August 2 to December 6, 2021. Omicron BA.1 (or B.1.1.529.1) became the dominant variant between December 20, 2021 and February 28, 2022, and Omicron BA.2 (or B.1.1.529.2) between March 14, 2022 and May 23, 2022. The currently circulating variants of interest SARS‐CoV‐2 BA.2.86 has inherited more than 30 mutations in its spike protein.

**Figure 1:**
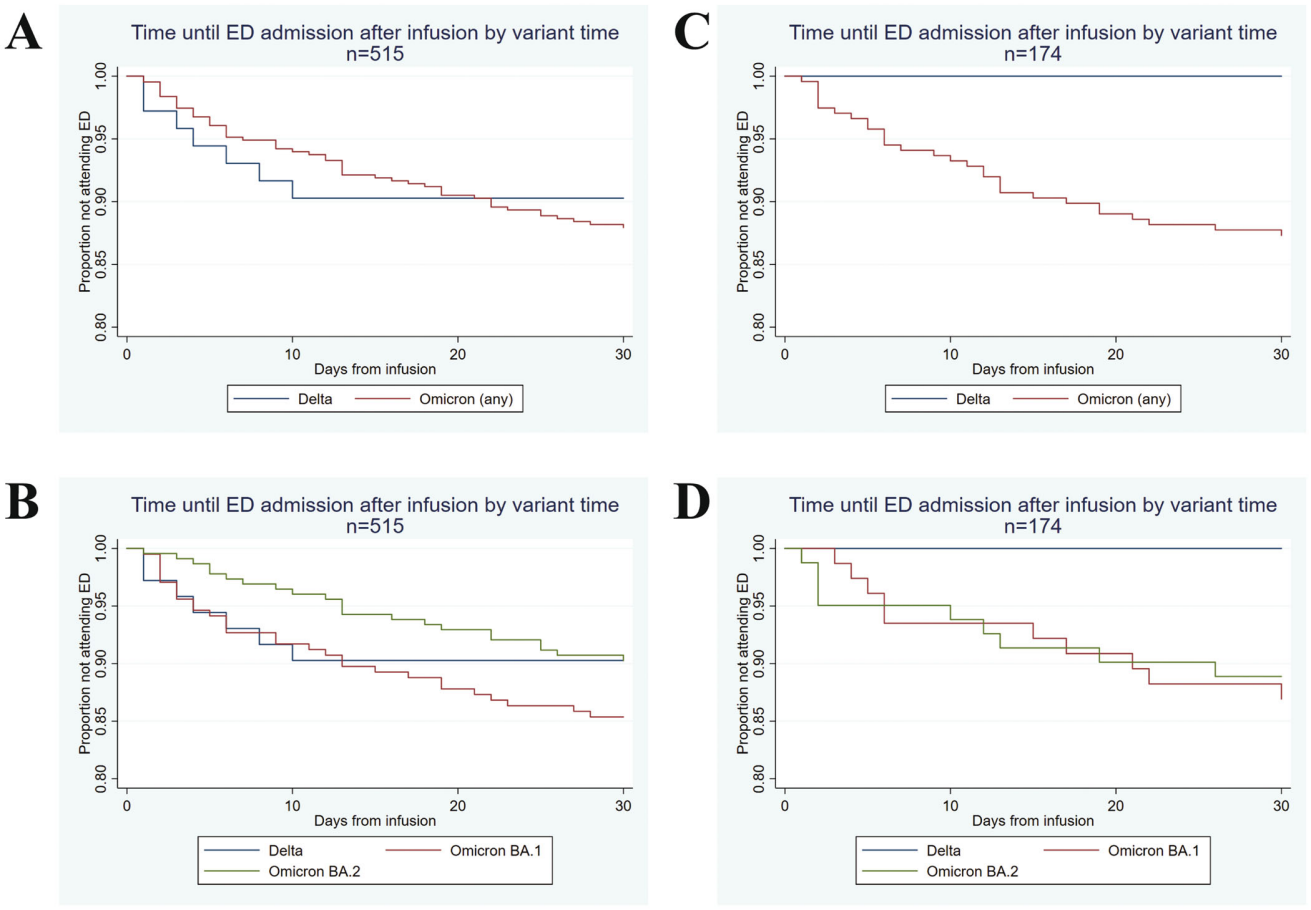
Effectiveness of sotrovimab for the treatment of SARS‐CoV‐2 infection during Omicron BA.2 subvariant predominance in Western Sydney, Australia. Unadjusted Kaplan–Meier curves for 30‐day risk of emergency department visit stratified by SARS‐CoV‐2 variant (A) subvariant (B) estimated based on date of infection (*n* = 515). Unadjusted Kaplan–Meier curves for 30‐day risk of ED Visit stratified by SARS‐CoV‐2 variant (C) subvariant (D) identified by sequencing (*n* = 174).

To assess COVID‐19‐related ED presentation and admissions, patients were stratified by variant and compared using standard Kaplan–Meier plots. Sotrovimab appeared to have similarly clinical efficacy at preventing ED presentations for both Delta and Omicron variants (Figure 1A) and subvariants (Figure 1B) upon assigning variant status based on the date of infection. In an uncorrected Cox regression model, there was a minor but nonsignificant increase in risk for Omicron patients to attend ED compared to Delta patients, with a hazard ratio (HR) of 1.22 (0.56–2.70). After correction, no difference in risk was identified (HR 0.97, 0.36–2.64) was noted. Comparison of Delta and Omicron variants (Figure 1C) and subvariants (Figure 1D) confirmed by sequencing (*n* = 174) was performed but was limited by the low number of Delta samples sequenced (*n* = 12). Nonetheless, Omicron subvariant status (BA.1 vs. BA.2) did not affect ED admission.

## 4 Discussion

Therapies for COVID‐19 that maintain activity even in the face of a rapidly evolving virus are crucial. Our real‐life data suggests that sotrovimab remains clinically effective against SARS‐CoV‐2 Delta, Omicron BA.1 and BA.2 variants. This is contrary to in vitro studies that have demonstrated significantly reduced neutralisation of Omicron variants, particularly BA.2 [10, 12]. Furthermore, this real‐world data indicates that sotrovimab was as effective at preventing severe clinical outcomes, such as early ED presentation and subsequent hospitalization, for all three variants. These data are consistent with previous reports finding that sotrovimab improved high risk patient survival in periods of Delta and Omicron variant predominance [13]. Moreover, sequenced BA.2 infection did not appear to pose any additional risk of hospitalisation as compared to BA.1 in a large cohort study (*n* = 8850) of English patients prescribed sotrovimab [14].

Sotrovimab has demonstrated continued efficacy as new SARS‐CoV‐2 variants arise. In vitro findings have demonstrated neutralisation capacity against Omicron BA.1, BA.5, BQ.1.1, XBB and XBB.1.5 variants and ADCC targeting BQ.1.1 and XBB.1.5 [15, 16]; findings that were not replicated using other mAbs such as imdevimab/casirivimab or cilgavimab/tixagevimab. Compared to untreated patients, Sotrovimab reduced hospitalisation and mortality across four studies during the period of Omicron BA.2 predominance (reviewed in [17]). Findings outlining sotrovimab efficacy compared to untreated controls during periods of BA.5 predominance are lacking, however there appears to be no difference in hospitalisation among sotrovimab treated patients during periods of BA.1, BA.2 and BA.5 predominance [18]. Similarly, sotrovimab was as effective as Paxlovid at reducing hospitalisation and death during the period of BA.2 and BA.5 predominance [19].

As an alternative to Sotrovimab, antivirals such as Paxlovid or Molnupiravir result in similar patient outcomes [19], but do not require hospital‐facilitated infusions and can be taken orally from home. While there are key benefits to oral antivirals, Molnupiravir has been shown to increase viral mutation rates and could therefore contribute to the development of future variants of concern with increased health burden [20]. Paxlovid is contraindicated with numerous drugs metabolised through similar pathways and may therefore be less suitable among vulnerable populations. With significantly different mechanisms of action, combining oral antivirals and mAb‐based therapies will nonetheless limit SARS‐CoV‐2 evolution whilst improving patient outcomes, particularly among the immunocompromised [21].

We acknowledge some limitations with our study: The data was collected during a pandemic and hence, not all patients could be tested for subvariants. Beyond screening questions by the population health team to ascertain eligibility for mAb therapy, we were unable to establish the precise time from symptom onset to the start of treatment from the data we obtained and thus cannot infer conclusions involving delayed or accelerated treatment. Further, the rate of hospitalisation was not examined among patients who did not receive sotrovimab, limiting the analysis of sotrovimab efficacy as compared to an untreated population. Lastly, our analysis was limited by the size of the sequenced SARS‐CoV‐2 Delta variant patient cohort (*n* = 12), with no patients having attended ED 30 days post sotrovimab infusion.

Finally, our findings underscore the importance of stewardship of mAbs in the fight against COVID‐19, particularly sotrovimab, as it has the potential to remain therapeutically relevant and effective though in disparate manner for the different Omicron sub‐lineages and potentially against emerging novel variants of concerns. The recent variant of interest, a descendant of BA.2.86, JN.1 (BA.2.86.1.1), has acquired an additional new receptor binding domain mutation (Leu455Ser) and other three mutations in non‐spike proteins compared to its predecessor BA.2.86, conferring the competitive advantage of specifically higher transmissibility and capacity to escape immune responses [22]. Moreover, the evolution of SARS‐CoV‐2 has been shown to drive resistance to novel monoclonal antibodies and/or antivirals such as Paxlovid [23]. In an era where future pandemics are likely, and rapid access to in vitro models and testing is readily available, we need to remain vigilant not to translate research lab results from in vitro testing into clinical practice. Especially, when there is a paucity of therapeutic agents available, it is essential not to withhold a treatment when its ineffectiveness has not been clinically proven. As our data does not support a reduction in real‐world clinical effectiveness of sotrovimab between variants, sotrovimab should be accessible to patients at high risk of severe SARS‐CoV‐2 disease.

In conclusion, in vitro results do not necessarily reflect in vivo clinical outcomes. Given the likelihood of future pandemics from SARS, and other organisms, our findings highlight an imperative lesson to be carried forward into the future about the potential of repurposing mAb treatments for future outbreaks.

## Author Contributions

E.K., Z.B., S.R., J.M., and G.A. contributed to the study concept and design. Z.B., V.K., L.W., and H.C. acquired clinical data. E.K., Z.B., G.M.K., V.K., S.R., M.O., J.E. and G.A. participated in the analysis and interpretation of the data. E.K. drafted the manuscript. All authors critically revised the manuscript for important intellectual content. G.A. and S.R. provided supervision. All authors approved the final version of the article, including the authorship. V.K. data collection and interpretation.

## Ethics Statement

The corresponding author, on behalf of all authors, jointly and severally, certifies that their institution has approved the protocol for any investigation involving humans or animals and that all experimentation was conducted in conformity with ethical and humane principles of research. The study was approved by the human research ethics committee of Western Sydney Local Health District (WSLHD) (Approval Number 2022/ETH00507).

## Conflicts of Interest

The authors declare no conflicts of interest.

## Supporting information

Supporting information.

Supporting information.

Supporting information.

Supporting information.

Supporting information.

Supporting information.

## Data Availability

The data that support the findings of this study are available on request from the corresponding author. The data are not publicly available due to privacy or ethical restrictions.
