# Supplementary figures and images for: Real‐World Effectiveness of Sotrovimab in Patients Infected With SARS‐CoV‐2 Omicron Subvariant BA.2 in Western Sydney, Australia
Source: J Med Virol. 2025 Feb 13;97(2):e70235. doi: 10.1002/jmv.70235 (PMC11822876; doi:10.1002/jmv.70235)

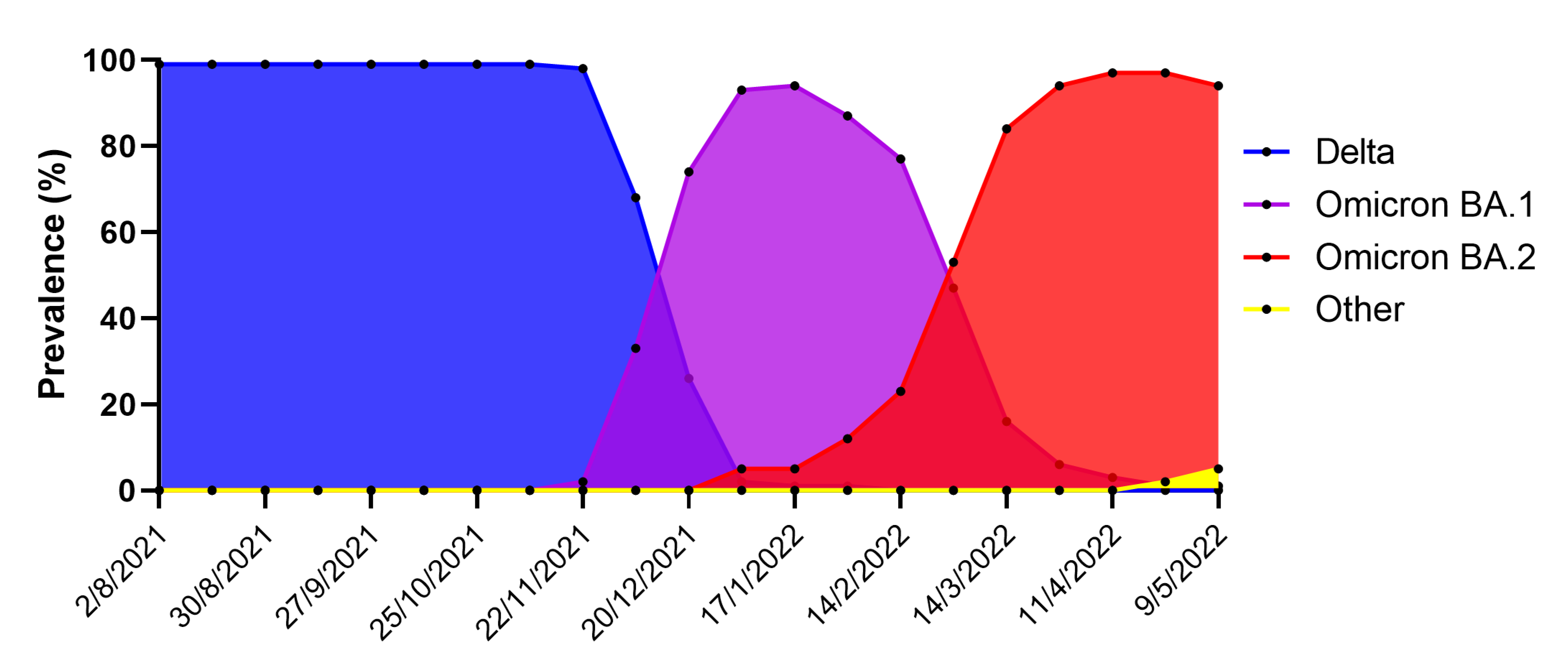

Supplement: Supplementary file 1 — Supporting information. [file JMV-97-e70235-s005.tif]
