# Supplementary material for: Real‐World Effectiveness of Sotrovimab in Patients Infected With SARS‐CoV‐2 Omicron Subvariant BA.2 in Western Sydney, Australia
Source: J Med Virol. 2025 Feb 13;97(2):e70235. doi: 10.1002/jmv.70235 (PMC11822876; doi:10.1002/jmv.70235)
Supplement: Supplementary file 4 — Supporting information. [file JMV-97-e70235-s003.docx]

| **Characteristic** | **Un-sequenced** | **Delta** | **OmicronBA1** | **OmicronBA2** |
| --- | --- | --- | --- | --- |
| All | (n=261) | (n=12) | (n=81) | (n=81) |
| Age | 51.1 (16.7) | 42.6 (17.0) | 52.1 (14.6) | 51.4 (16.7) |
| Sex |  |  |  |  |
| *Female* | 147 (56.3%) | 4 (33.3%) | 37 (45.7%) | 40 (49.4%) |
| *Male* | 114 (43.7%) | 8 (66.7%) | 44 (54.3%) | 41 (50.6%) |
| Vaccine Status |  |  |  |  |
| *Unvaccinated* | 35 (13.4%) | 5 (41.7%) | 7 (8.6%) | 5 (6.2%) |
| *One dose* | 31 (11.9%) | 5 (41.7%) | 1 (1.2%) | 1 (1.2%) |
| *Two doses* | 93 (35.6%) | - | 36 (44.4%) | 34 (42.0%) |
| *Three doses* | 91 (34.9%) | 2 (16.7%) | 35 (43.2%) | 37 (45.7%) |
| *Four doses* | 10 (3.8%) | - | 2 (2.5%) | 4 (4.9%) |
| Comorbidities |  |  |  |  |
| *Cardiac* | 35 (13.4%) | 3 (25.0%) | 13 (16.0%) | 7 (8.6%) |
| *Diabetes* | 51 (19.5%) | 4 (33.3%) | 20 (24.7%) | 20 (24.7%) |
| *Chronic Lung Disease* | 30 (11.5%) | 2 (16.7%) | 8 (9.9%) | 10 (12.3%) |
| *Kidney Disease* | 52 (19.9%) | 2 (16.7%) | 29 (35.8%) | 14 (17.3%) |
| *Liver Disease* | 2 (0.8%) | - | 3 (3.7%) | 1 (1.2%) |
| *Cancer* | 30 (11.5%) | 1 (8.3%) | 14 (17.3%) | 16 (19.8%) |
| *Immunocompromised* | 149 (57.1%) | - | 55 (67.9%) | 57 (70.4%) |
| *Obese (BMI>30)* | 76 (29.1%) | 9 (75.0%) | 16 (19.8%) | 10 (12.3%) |
| ED Admission | 34 (13.0%) | 1 (8.3%) | 12 (14.8%) | 10 (12.3%) |
| Death | 1 (0.4%) | - | - | - |

**Table S1**. Baseline characteristics of SARS-CoV-2 patients where variant of infection was confirmed by sequencing.
