# Supplementary material for: Real‐World Effectiveness of Sotrovimab in Patients Infected With SARS‐CoV‐2 Omicron Subvariant BA.2 in Western Sydney, Australia
Source: J Med Virol. 2025 Feb 13;97(2):e70235. doi: 10.1002/jmv.70235 (PMC11822876; doi:10.1002/jmv.70235)
Supplement: Supplementary file 5 — Supporting information. [file JMV-97-e70235-s002.docx]

**Table S2.** Clinical criteria for Sotrovimab administration and prioritised cohorts in NSW.

**Clinical criteria for Sotrovimab administration in NSW**

As per the National Taskforce Guidelines, sotrovimab is appropriate for use in adult patients:

• within five (5) days of symptom onset (symptoms may be very mild); AND

• who do not require oxygen; AND

• who have not been fully vaccinated (note: fully vaccinated means 2nd dose > 2 weeks ago); AND

• who have one or more of the following risk factors for disease progression;

−diabetes (requiring medication)

−obesity (BMI > 30 kg/m2)

−chronic kidney disease (i.e. eGFR < 60 by MDRD)

−congestive heart failure (NYHA class II or greater)

−chronic obstructive pulmonary disease (history of chronic bronchitis, chronic obstructive lung disease, or emphysema with dyspnoea on physical exertion)

−moderate-to-severe asthma (requiring an inhaled steroid to control symptoms or prescribed a course of oral steroids in the previous 12 months)

−age ≥ 55 years.

The following additional risk factors should also be considered:

• patients who are immunosuppressed, even if they are partially or fully vaccinated

• Aboriginal and/or Torres Strait Islander patients > 35 years old.

Clinical judgement should be used when assessing the severity of specific risk factors. This may include other significant chronic health conditions including but not limited to cardiac failure, chronic lung disease, immunosuppression, and active malignancy.

**Prioritised cohorts in NSW**

Access for patients should be considered in the context of the outbreak in NSW. As such, it is the recommendation of the clinical working group that the following cohorts are prioritised. Patients identified as part of the following groups also need to meet the clinical criteria above.

• Aboriginal and/or Torres Strait Islander communities.

• Rural, regional, and remote communities: a. Where there is a significant outbreak impacting the community.b. For patients who have been brought to a regional centre (from a remote location) for monitoring due to their risk of acquiring severe disease. c. For patients who are located in a remote location and prefer to remain in their community and/or on country.

• To support the public health response in metropolitan areas with large outbreaks.

• Nosocomial patients – those who have acquired a COVID-19 infection in hospital or healthcare setting.

• Patients who have acquired COVID-19 infection in high risk settings such as disability group homes and residential aged care facilities. It may be prudent to plan access for patients in the above groups who have been exposed but have not yet developed symptoms.
