# Supplementary material for: Real‐World Effectiveness of Sotrovimab in Patients Infected With SARS‐CoV‐2 Omicron Subvariant BA.2 in Western Sydney, Australia
Source: J Med Virol. 2025 Feb 13;97(2):e70235. doi: 10.1002/jmv.70235 (PMC11822876; doi:10.1002/jmv.70235)

**Table S3.** Cox regression analysis of SARS-CoV-2 variants identified by time of infection (A) or sequencing (B).

**A**


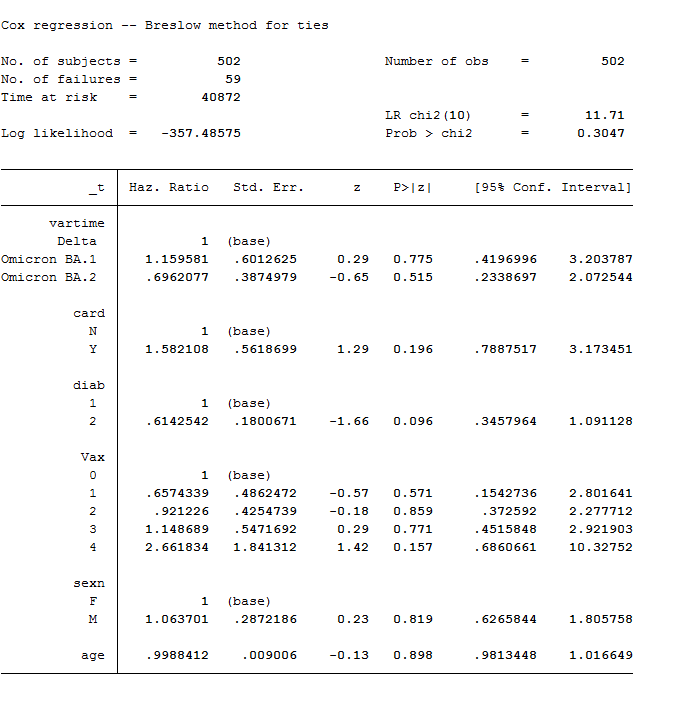


**B**


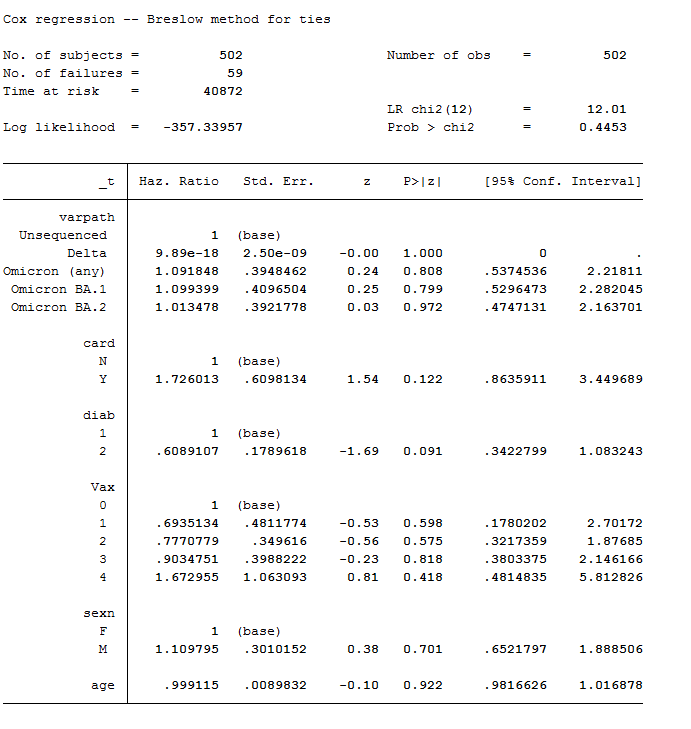

Supplement: Supplementary file 6 — Supporting information. [file JMV-97-e70235-s006.docx]
